# IL-27, IL-30, and IL-35: A Cytokine Triumvirate in Cancer

**DOI:** 10.3389/fonc.2019.00969

**Published:** 2019-10-01

**Authors:** Olena Kourko, Kyle Seaver, Natalya Odoardi, Sameh Basta, Katrina Gee

**Affiliations:** Department of Biomedical and Molecular Sciences, Queen's University, Kingston, ON, Canada

**Keywords:** cytokines, signaling, immunotherapy, tumor microenvironment, cancer

## Abstract

The role of the immune system in anti-tumor immunity cannot be overstated, as it holds the potential to promote tumor eradication or prevent tumor cell escape. Cytokines are critical to influencing the immune responses and interactions with non-immune cells. Recently, the IL-12 and IL-6 family of cytokines have accumulated newly defined members each with specific immune functions related to various cancers and tumorigenesis. There is a need to better understand how cytokines like IL-27, IL-30, and IL-35 interact with one another, and how a developing tumor can exploit these interactions to enhance immune suppression. Current cytokine-based immunotherapies are associated with cytotoxic side effects which limits the success of treatment. In addition to this toxicity, understanding the complex interactions between immune and cancer cells may be one of the greatest challenges to developing a successful immunotherapy. In this review, we bring forth IL-27, IL-30, and IL-35, “sister cytokines,” along with more recent additions to the IL-12 family, which serve distinct purposes despite sharing structural similarities. We highlight how these cytokines function in the tumor microenvironment by examining their direct effects on cancer cells as well their indirect actions via regulatory functions of immune cells that act to either instigate or inhibit tumor progression. Understanding the context dependent immunomodulatory outcomes of these sister cytokines, as well as their regulation within the tumor microenvironment, may shed light onto novel cancer therapeutic treatments or targets.

## Introduction

Cytokine production in the tumor microenvironment dictates how innate and adaptive immune cells respond to and influence tumor progression. Irregular cytokine production by tumor cells and immune cells within the tumor microenvironment (TME) can be used as a diagnostic tool to screen patients for cancer (1–3). Cytokines are master regulators of inflammatory cell function and can recruit, activate, or dampen immune responses, in addition to regulating cellular proliferation and differentiation (4, 5).

**Figure 1:**
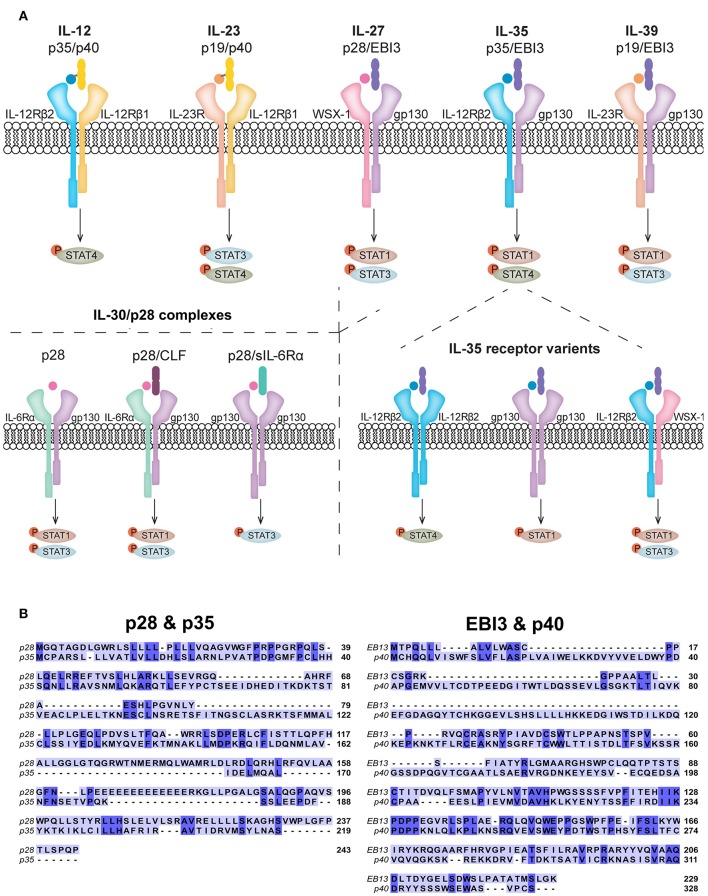
Receptor composition of IL-12 family members and related cytokines. **(A)** The IL-12 family members are heterodimeric in composition and signal via heterodimeric receptors. IL-27, composed of p28 and EBI3, shares common subunits with IL-35 (p35 and EBI3) and IL-39 (p19 and EBI3). The p28 subunit has also been shown to have biological activity in the absence of EBI3, known as IL-30. In addition to subunit sharing with IL-27 and IL-39, IL-35 shares the p35 subunit with IL-12. p28 is also known to form complexes with CLF and sIL-6Rα. Signaling of all IL-12 family members, IL-30, and other known p28 complexes, predominantly activate the JAK/STAT pathway. Each cytokine preferentially activates specific STATs to achieve its functional role, although other phosphorylated STATs have been observed. **(B)** Amino acid sequence overlays for human p28 (accession number: NP_663634.2) with p35 (accession number: NP_000873.2) and EBI3 (accession number: NP_005746.2) with p40 (accession number: NP_002178.2) were aligned using the T-COFFEE-espresso program (11). Shared amino acids are indicated by dark blue shading and homology between p28 and p35 was calculated to be 64% (44 of 169) and between EBI3 and p40 was calculated to be 33% (59 of 177).

Due to the complex nature of the TME it is important to understand the role cytokines play in influencing tumor and immune cell interactions (1, 5). Two prominent type I cytokine families, IL-6 and IL-12, have been a recent focus in cancer research. The IL-6 family of cytokines includes IL-6, IL-11, IL-27, IL-31, leukemia-inhibitory factor (LIF), cardiotropin-1 (CT-1), oncostatin M (OSM), ciliary neurotrophic factor (CNTF), and cardiotropin-like cytokine (CLC), which share the glycoprotein 130 (gp130) receptor subunit (6). As prominent factors in several essential cellular processes, IL-6 family members are considered pro-tumorigenic (7, 8), with the exception of IL-27, which has a more complex role in potential anti-tumor therapeutic implications (9). Adding to this complexity, the newly identified IL-30 cytokine, which shares a subunit with IL-27, promotes tumor progression (10). IL-27 also belongs to the IL-12 family, which includes IL-12, IL-23, IL-27, IL-35, and IL-39 (Figure 1A). Although IL-35 is considered to belong to the IL-12 family, due to its usage of the gp130 receptor it is also related to members of the IL-6 family (12). The ability of IL-12 to activate anti-tumor cytotoxic T lymphocytes (CTLs) and natural killer (NK) cells, makes it an attractive cytokine for cancer immunotherapy (13). Despite overwhelming evidence toward the anti-tumor effects of IL-12, its use in the clinic remains problematic due to toxicity concerns (14–16). Cytokines closely related to IL-12 are being investigated for their anti-tumor capacity, and of these cytokines IL-27 has become an attractive alternative based on its potent anti-tumor activity (17). Here, we review the potential interplay of the three sister cytokines; IL-27, IL-30, and IL-35, and their role in tumorigenesis.

## Overview of IL-27, IL-30, and IL-35 Signaling

### IL-27

Discovered in 2002, IL-27 is composed of two non-covalently linked subunits, IL-27p28 (p28) and Epstein-Barr-virus-induced molecule 3 (EBI3) (18) (Figure 1A). These subunits exhibit structural and sequence homology to IL-12 subunits and IL-6 (19). The p28 subunit shares ~64% amino acid homology with the p35 subunit of IL-12, based on their sequence alignment (Figure 1B). p28 also shares ~27% homology with IL-6 (20, 21). EBI3 shares ~33% amino acid homology with IL-12p40 based on their sequence alignment (Figure 1B) and in agreement with Devergne et al. (22) who calculated 27% homology. IL-27 binds a heterodimeric receptor comprised of gp130 and WSX-1 (23). WSX-1 is highly expressed on lymphocytes and at lower levels on myeloid cells (23, 24). Gp130 is ubiquitously expressed, therefore the specificity of IL-27 activity is determined by co-expression of WSX-1 (25). IL-27 binds WSX-1 with low affinity in the absence of gp130 (18, 23), however, both receptor subunits are required for efficient signaling (23). In addition, WSX-1 and gp130 exist in soluble forms (sWSX-1 and sgp130) and binding of sWSX-1 to IL-27 acts as a signaling antagonist (26). Interestingly, sgp130 does not inhibit IL-27 signaling (27), although it does block IL-11 and IL-6 trans-signaling (28). This indicates the possibility that sgp130 exclusively inhibits gp130 homodimer signaling. Upon IL-27 binding to its receptor, phosphorylation of signal transducer and activator of transcription (STAT) 1 and 3 occurs, which is negatively regulated by suppressor of cytokine signaling 3 (SOCS3) (23, 29). The cell type and activation state may influence IL-27 signaling to modify comparative levels of STAT activation (30, 31).

IL-27 is produced by antigen presenting cells (APCs) following Toll-like receptor (TLR) activation (18, 32, 33). Initially, IL-27 was characterized as a positive mediator of Th1 development (18, 34). Alternatively, IL-27 also demonstrates anti-inflammatory and inhibitory properties, particularly with regards to inhibition of Th2, Th17 and Treg differentiation (35, 36). However, more recent studies have contrasted these results, indicating that IL-27 can promote the growth and survival of Treg cells (37, 38).

IL-27 produced in response to TLR activation also modulates myeloid cell function and results in upregulation of TLR expression on these cells (21, 39–42). This indicates that IL-27 may participate in a positive feedback loop, whereby activated cells express IL-27 which then acts to further sensitize cells to TLR responsiveness. IL-27-treated myeloid cells and epithelial cells also exhibit enhanced antigen presentation by upregulation of major histocompatibility complex (MHC) I and II as well as co-stimulatory molecules (43–45). Due to its ability to affect antigen presentation and modulate helper T cell differentiation and activation, IL-27 holds the potential to be a master regulator in the TME.

### IL-30

Originally identified as a subunit of IL-27, p28 alone, known as IL-30, can exert biological activities independently from EBI3 (21, 46–49). Sharing homology with IL-6 family members: IL-6, IL-11, and CLC (18), p28 can function as an antagonist of gp130 signaling, blocking IL-6, IL-11, and IL-27 functions (50). Various p28 binding partners have been identified to induce signaling, these include CLF, p40, and sIL-6Rα (51). Recent reports showed that IL-30 signals similarly to IL-6, by interacting with CLF or soluble IL-6Rα and membrane-bound gp130 homodimers, resulting in phosphorylation of STAT1 and STAT3 (21, 51, 52) (Figure 1A). Interestingly, IL-30 at high concentrations can also signal through gp130 homodimers in the absence of soluble IL-6Rα (52). Furthermore, in human monocytes, recombinant IL-30 treatment resulted in delayed STAT1 and STAT3 phosphorylation compared to that induced by IL-27; the delay in IL-30-mediated STAT phosphorylation correlated with levels of sIL-6Rα expression, further supporting a role for IL-6Rα in IL-30 signaling (21).

IL-30 production measured by detection of p28 mRNA or secreted protein levels can be detected in activated macrophages and splenocytes (53). Furthermore, recombinant IL-30 acts on monocytes to induce IFN-γ-inducible protein 10 (IP-10; CXCL10) and TLR4 expression (21). Recent evidence supports a protective role of IL-30 against liver injury and fibrosis (46). It is important to consider which cells specifically express and/or respond to p28 and its potential binding partners which can exert supplementary functions compared to IL-30 (p28 alone). For example, the p28/CLF complex is produced by activated DCs and modulates NK cell and T cell function (51) and enhances proliferation and differentiation of plasma cells (54). The existence of different p28 binding partners implies that IL-30, either as p28 alone or in combination with one or more partners, may result in differential functions depending on cell type and activation state.

### IL-35

IL-35 was discovered in 2007 and, similar to other IL-12 family members, is a heterodimeric cytokine comprised of the IL-12p35 (p35) and EBI3 (55, 56). The IL-35 receptor components vary by cell type; in T cells, IL-35 binds gp130 and IL-12Rβ2 to signal through either gp130/IL-12Rβ2 heterodimers or homodimers of each subunit (57, 58). Alternatively, in B cells, IL-35 signals through IL-12Rβ2 and WSX-1 (59). Since IL-35 can signal through gp130 and/or WSX-1, this indicates the potential for competition for receptor usage between IL-27, IL-30 and IL-35. Depending on the receptor chains that are engaged, IL-35 elicits activation of various STATs (Figure 1A). For example, IL-35 binding to IL-12Rβ2:WSX-1 results in STAT1 and STAT3 phosphorylation (59), while binding to IL-12Rβ2:gp130 induces formation of a STAT1:STAT4 heterodimer. IL-35 signaling via IL-12Rβ2 or gp130 homodimers results in STAT4 or STAT1 phosphorylation, respectively (58).

Secreted primarily by Treg cells in response to either IFN-γ or TLR stimulation, IL-35 opposes pro-inflammatory functions of other IL-12 family members (55). Cell types such as B cells, endothelial cells, smooth muscle cells, DCs, and monocytes also produce IL-35, but to lesser extent than Tregs (59–65). IL-35 converts T cells and B cells into regulatory populations that produce IL-35, referred to as inducible Treg-IL-35 (iTr35) and IL-35^+^ Breg cells (60, 64). IL-35 inhibits differentiation and function of Th1 and Th17 cells by promoting the expansion of Tregs and their production of IL-10 (55, 60). IL-35 is an important cytokine with immunosuppressive functions, which play a role in promoting a pro-tumor phenotype within the TME. Competition between IL-27, IL-30, and IL-35 for receptor subunits may affect the overall balance of pro- vs. anti-tumor effects of these cytokines. The differential roles of each cytokine in cancer is discussed below.

## IL-27, IL-30, and IL-35: Influencing Cancer Development

### IL-27: The Cytokine That Swings Both Ways

**Figure 2:**
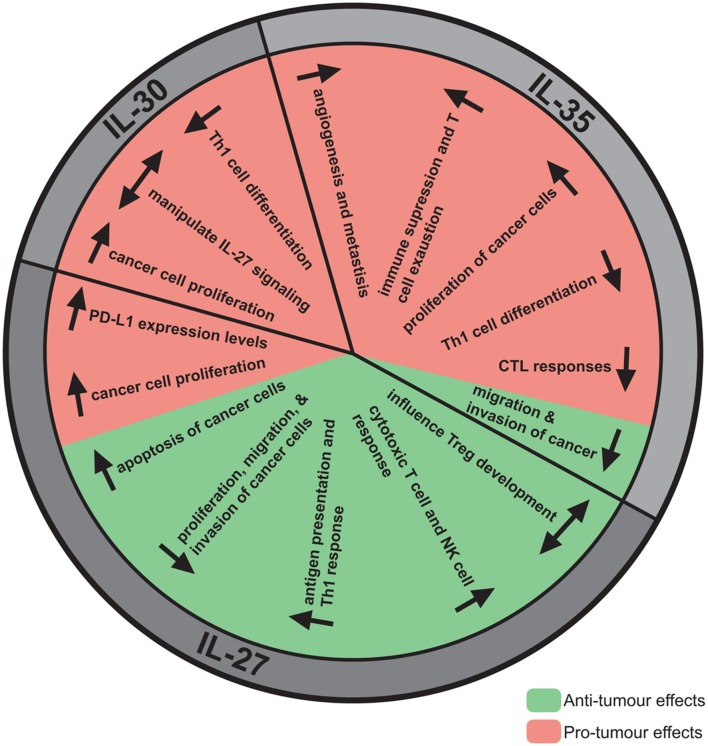
The anti- and pro-tumor effects of IL-27, IL-30, and IL-35. Although IL-27, IL-30, and IL-35 share subunits, these cytokines have direct and indirect effects on the tumor resulting in either tumor progression or elimination. IL-27 has mainly been demonstrated to have anti-tumor effects, most notably decreasing proliferation, migration, and invasion, enhancing apoptosis, and promoting cytotoxic immune responses. Pro-tumor effects have also been observed for IL-27, such as upregulation of PD-L1. Alternatively, IL-30 has not been studied extensively but pro-tumor effects have been identified, such as promoting cancer cell proliferation, and decreasing Th1 differentiation. IL-35 has been implicated in promoting tumor advancement by increasing cancer cell proliferation, angiogenesis, metastasis, immune suppression, and T cell exhaustion. Contrastingly, IL-35 may have anti-tumor effects attributed to its potential role in decreasing cancer cell migration and invasion.

**Figure 3:**
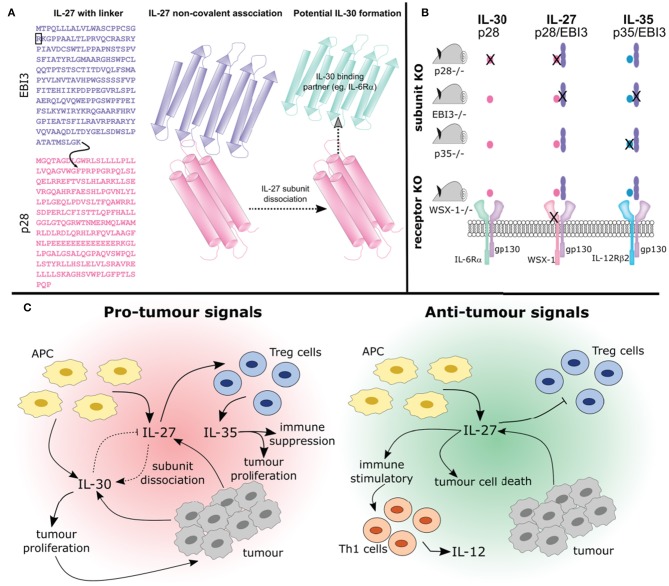
Studying the interplay between IL-27, IL-30, and IL-35. **(A)** The synthesis of IL-27 as a purified recombinant protein or transduced expression vector varies. Both of these forms of IL-27 are available in two formats: (1) containing a flexible amino acid linker sequence (indicated by the curved black arrow), that joins the EBI3 subunit lacking its signal sequence (indicated by the black box) to phenylalanine 29, after the signal sequence of p28 (**A**; left) or (2) the two subunits co-expressed which associate non-covalently (**A**; middle). Thus, engineered IL-27 may differ from its endogenously expressed counterpart whereby the flexible amino acid linker prevents the possibility of subunit dissociation. Furthermore, whether non-covalently associated IL-27 subunits can dissociate to form IL-30 (i.e., the p28 subunit) or if they associate with another binding partner is not known (**A**; right). **(B)** Studying the functions of cytokines using knockout mice is complex and the outcomes should be carefully considered. Using p28 knockout mice will result in IL-30 and IL-27 elimination, whereas knockout of p35 eliminates IL-35 and IL-12 (not depicted). Knockout of EBI3 removes both IL-27 and IL-35 (IL-39 is also removed, not shown). Using a WSX-1 receptor chain knockout will prevent IL-27 signaling and may prevent signaling of IL-30. Additionally, IL-35 signaling on B cells will be inhibited (not shown). **(C)** How these cytokines interact will influence tumor development. The pleiotropic effects of IL-27 produced by APCs can be seen here. IL-27 can promote differentiation of Treg cells which secrete IL-35 resulting in immune suppression and tumor development. Alternatively IL-27 can prevent Treg development and promote anti-cancer Th1 cell development. Importantly, IL-27 may undergo subunit dissociation giving rise to the pro-tumor cytokine IL-30, or can directly act on cancer cells resulting in apoptosis. Overall, the complex relationships between IL-27, IL-30, and IL-35 need to be considered when discussing their potential role in cancer immunity.

Recently, both anti-tumor and pro-tumor roles have been attributed to IL-27 (Figure 2). This dichotomy may be explained, in part, by the differential roles played by IL-27 in the immune response. The roles for IL-27 in cancer have been studied in both *in vitro* and *in vivo* models and it is important to consider how IL-27 is introduced to the model system given that this cytokine is heterodimeric and the subunits are non-covalently associated in nature. Commercially available recombinant IL-27 and IL-27 expression vectors may contain an engineered flexible amino acid linker sequence between EBI3 and p28 subunits, potentially preventing subunit dissociation and thus formation of IL-30 or IL-35 (Figure 3A). While several studies examine both recombinant and transduced IL-27, caution should be considered when interpreting data from studies where the linker in synthetic IL-27 is used because its presence or absence has yet to be directly compared and assessed. By treating cells *in vitro* with recombinant cytokine, the dose, cell number, and length of exposure to a specific cell type can be defined, where these parameters are more difficult to control in an *in vivo* model. *In vivo* studies using cancer cells transduced with an IL-27 expression vector permits continual IL-27 production and ensures that IL-27 is present within the TME; however, the dose and length of exposure becomes more challenging to control in the studied model. When taking into account the use of knockout animals, it is important to acknowledge that deficiency in cytokine or receptor subunits may impact more than one particular cytokine as outlined in Figure 3B.

#### Anti-tumor Impact of IL-27

IL-27 can directly suppress tumorigenesis by interacting with cancer cells, or indirectly by stimulating different subsets of immune cells resulting in their activation against a developing tumor. The direct anti-tumor effects of IL-27 results from its ability to suppress cancer cell proliferation, migration, and invasion, and enhance cancer cell death (66–70). For example, IL-27 has direct anti-proliferative effects on both mouse and human melanoma cells in a STAT1 dependent manner (71). IL-27 also directly induces apoptosis in prostate cancer cells, multiple myeloma, non-small cell lung cancer, and ovarian cancer cells (72–75). Furthermore, IL-27 has the potential to interact with novel therapeutics; it synergizes with poly(I:C), a TLR3 agonist, to reduce proliferation of melanoma and prostate cancer cells via TRAIL or TLR3, respectively (76, 77). IL-27 upregulates TLRs in cancer cells and immune cells, resulting in enhanced cellular responsiveness to TLR agonists, supporting the combination of IL-27 and TLR agonists as a potential cancer therapy (39–42, 76–79).

Additionally, IL-27 can promote anti-tumor immune responses by altering development and activation of various T cell subsets and NK cells. CTLs specifically target and eliminate cancer cells expressing a tumor associated antigen (80–84). IL-27 enhances the development, proliferation, and cytotoxic activity of CD8^+^ T cells, thereby indirectly promoting anti-tumor immunity (85). In *in vitro* and *in vivo* models, the use of either recombinant IL-27 or IL-27-transduced cancer cell lines has characterized the role of IL-27 in regulation of the CTL response. Using a colon cancer cell line (C26) transduced with IL-27, Hisada et al. demonstrated that IL-27 promoted C26-specific CD8^+^ T cell activity resulting in a robust anti-tumor immune response (17). Furthermore, IL-27 enhances anti-tumor CD8^+^ T cell survival (86). Schreider et al. observed a similar role for IL-27 acting directly on CD8^+^ human T cells and driving cytotoxic activity (87). The use of mice deficient in WSX-1, EBI3, or p28 also suggest a role for IL-27 in promoting anti-tumor CD8^+^ T cell responses (88–91). IL-27 is also involved in the development of memory CD8^+^ T cell responses (89). Taken together, these studies present IL-27 as an anti-tumor cytokine capable of promoting tumor antigen specific CTL development, enhancing their survival, and differentiation into memory effector T cells.

CD4^+^ T cells play an important role in the TME and can contribute to an anti-tumor immune response. One of the reasons IL-12 demonstrated success as an anti-tumor agent was in part due to its ability to promote a Th1 immune response (92). Similarly, IL-27 can act on naive CD4^+^ T cells to influence their differentiation into Th1 cells and establish enhanced CD4^+^ T cell responsiveness to IL-12 (18, 35). On the other hand, IL-27 prevents GATA-3 expression, resulting in reduction of Th2 polarization (35, 93, 94). Since IL-27 regulates the balance of Th1 and Th2 cells, it has the potential to skew the CD4^+^T cell response toward Th1, which could enhance tumor regression. IL-27 also inhibits the development and function of Treg cells, which suppress T cell activation in the TME permitting tumor cell escape (95–98). Therefore, targeting Tregs and preventing their differentiation, serves as an important consideration in the development of successful cancer immunotherapies. Using a recombinant adenovirus vector expressing IL-27, Zhu et al. demonstrated that IL-27 decreases Treg cell numbers, while increasing IL-10 to prevent excessive inflammation (99). Taken together, IL-27 has important implications in the development of an anti-tumor T cell response by enhancing CTL activation, promoting Th1 mediated anti-tumor immune responses, and inhibiting Th2 and Treg mediated immune suppression.

In addition to having effects on T cells, IL-27 also influences macrophage polarization. Macrophages exist along a spectrum of phenotypes but can be generally classified as M1, predominantly exhibiting anti-tumor effects, or M2, generally displaying pro-tumor functions (100–102). Yao et al. observed that IL-27 was able to skew M2 polarized macrophages toward an M1 phenotype (103). Moreover, the presence of IL-27 reduced proliferation, migration, and invasion of pancreatic cancer cell lines co-cultured with M2 polarized macrophages (103). IL-27 was also able to sensitize the pancreatic cancer cells to gemcitabine, a chemotherapeutic drug, in the co-culture system. This suggests that IL-27 may influence the macrophage phenotype within the TME leading to modulation of tumor growth.

IL-27 also enhances NK cell cytotoxicity toward cancer cells (104–106). The ability of IL-27 to enhance NK cell mediated killing of cancer cells has been demonstrated in a series of murine models including hepatocellular carcinoma, melanoma, head and neck squamous cell carcinoma (107–110). These studies use either recombinant IL-27 or IL-27-transduced cancer cell lines to evaluate how IL-27 modulates NK cell function. In humans, the impact of IL-27 on NK cells in a tumor setting needs to be further investigated, in particular how IL-27 could simultaneously coordinate NK cell activity and tumor cell death.

#### Pro-Tumor Impact of IL-27

IL-27 has been implicated in promoting cancer progression, as it can promote proliferation of human leukemic cell lines (111). Furthermore, IL-27 can be secreted by tumor cells and high IL-27 levels are associated with advanced cancer (112–114). This association could be attributed to several ways in which IL-27 can influence the immune cells within the TME. As stated earlier, IL-27 directly induces apoptosis of cancer cells resulting in an anti-tumor immune response. However, these apoptotic cancer cells are endocytosed by DCs, which results in IL-27 expression leading to IL-27-dependent Treg activation (115). This concept is further supported by work in EBI3 and IL-27R KO mice demonstrating that IL-27 directly enhances Treg activity by inducing CD39 expression, resulting in tumor growth (116). Additionally, IL-27 stimulation induces PD-L1 expression on several types of cancer cells including: prostate, lung, and hepatocellular carcinoma (117, 118). PD-L1 expression is also induced by IL-27 in immune cell populations such as T cells and myeloid cells (119–121). It is important to consider the impact of IL-27 on PD-L1 expression as increased PD-L1 expression is associated with dampening CTL tumor-specific immune responses. IL-27 can also increase expression of CTLA4, LAG-3 and TIM-3 on T cells (122). There is evidence that combining IL-27 with an immune checkpoint inhibitor may enhance an immune response while simultaneously promoting the anti-tumor effects of IL-27, but further investigation is needed for these combination therapies (99). Taken together, IL-27 serves as an important modulating cytokine in the TME that can be used to either enhance the activation of potent anti-tumor immune cells, directly inhibiting cancer cell proliferation and angiogenesis or promote tumor development and survival (Figure 3C). Understanding how IL-27 is able to provide both immune stimulating and suppressing mechanisms in the TME is an important area of research requiring further investigation.

### IL-30: The Loss of Something Gives New Meaning

With the discovery that p28, without EBI3, can function independently as IL-30, new research has emerged to understand how IL-30 modulates immune responses in the TME and its role in tumorigenesis (Figure 2). IL-30 expression has been detected in human breast and prostate cancer tissue samples; administration of IL-30 in murine models enhanced tumor cell proliferation and migration (10, 123, 124). As an independent cytokine, IL-30 can inhibit gp130 signaling (50) in murine cells giving it the potential to negate the anti-tumor effects that IL-27 provides. The mechanisms involved in IL-30 production and its ability to function independently still need to be fully investigated. Understanding these mechanisms will provide insight into the factors involved in EBI3 and IL-27p28 engagement. By promoting this interaction, it may be possible to decrease the amount of independent p28 within the TME resulting in enhanced IL-27 activity, and in turn, a more robust anti-tumor immune response.

In addition to preventing IL-27 signaling, IL-30 can limit IL-12-induced Th1 differentiation (46), thus serving as an additional contributing factor to promoting tumor growth. Indeed, EBI3 KO mice have greater numbers of Treg cells (125). Using a B16-F10 malignant cell-line, Liu et al. demonstrated that s.c injection of B16-F10 cancer cells into EBI3 KO mice resulted in rapid tumor growth when compared to wild type (126). Since these studies use EBI3 KO mice, these mice are lacking IL-27 and IL-35, thus potentiating a role for IL-30 in promoting Treg development and contributing to tumor progression (Figure 3C). It is possible that IL-30 may inhibit the function of IL-27 in the event that both cytokines are co-expressed within the TME. With the limited understanding of IL-30 biology and specifically how it functions within the TME, more research is needed to fully elucidate the interplay between IL-27, IL-30, and IL-35 in cancer progression.

### IL-35: Different Partnership, Opposing Effect

It appears that IL-35, consisting of EBI3 associating with p35, rather than p28, brings a plethora of functions and responses that are generally contrasting that of IL-27. Early findings point to a role for IL-35 in promoting cancer development (Figure 2); expression of p35, but not p28, was correlated with EBI3 in Hodgkin lymphoma and nasopharengeal carcinoma (112, 127). In breast cancer, EBI3 levels positively correlate with p35, however no significant correlation between EBI3 and p28 was detected, implicating a role for IL-35 rather than IL-27 (128). The correlation between elevated levels of EBI3 and p35 rather than p28 may hold potential information to how a tumor is able to influence the TME cytokine profile to promote tumorigenesis. Others have shown high levels of EBI3 expression in other cancers, but these studies did not correlate EBI3 expression with p35 or p28 (127, 129, 130). Sauer et al. demonstrated that upon introduction of B16-F10 cells to EBI3 deficient mice, the mice were more resistant to lung metastasis (131); however, this study did not take into account the role of the other cytokine subunits. Currently, the general consensus is that IL-35 is positively correlated with increasing severity and stage of cancer. Using a combination of immunohistochemistry and/or IL-35-specific ELISA, several studies document increased levels of IL-35 in several cancers including: acute myeloid leukemia, colorectal cancer, pancreatic ductal adenocarcinoma (PDAC), clear cell renal carcinoma, breast invasive ductal carcinoma, and prostate carcinoma (132–138).

Like IL-27, expression of IL-35 can directly impact cancer cell survival by acting on tumor cells and due to its non-covalent heterodimeric nature, it is important to consider how IL-35 is delivered in the model system. Nicholl et al. found that recombinant IL-35 enhanced proliferation and survival of the pancreas adenocarcinoma cell line, MiaPaCA-2, in a dose-dependent manner (139). Furthermore, like IL-27, IL-35 influences immune responses in the TME. In both immune competent and Rag1/2-deficient mice, tumor cells engineered to express IL-35 exhibited increased myeloid cell accumulation in the TME resulting in increased tumor angiogenesis (140). This indicates that tumor-produced IL-35 is involved in promoting tumor survival. As well, immune competent mice lacked spontaneous CTL responses to tumors (140), thereby favoring tumor promoting conditions. This study showed that using monoclonal antibodies to neutralize IL-35, tumor development was abrogated. In addition to Tregs, Wang et al. demonstrated that IL-35 can also be produced by human cancer tissues (140). For instance, in breast and lung cancer models, TME macrophages secrete IL-35, which ultimately facilitated metastatic colonization of the cancer (141). Neutralization or knockout of IL-35 in the tumor associated macrophages (TAMs) reduced metastatic colonization (141). Similarly, the continual presence of IL-35 using an expression vector system *in vivo* can permit neutrophil infiltration into the TME and promote neutrophil polarization toward a pro-tumor phenotype (N2) (142). Together, these studies suggest that cancer cells as well as TAMs can be a source of IL-35, which function alongside neutrophils to contribute to immune suppression and enhanced tumorigenesis.

In human non-small-cell lung carcinoma (NSCLC) patients, IL-35 expression was increased in bronchoalveolar lavage fluid (BALF) from tumor sites. Upon exposure of purified CD4^+^ or CD8^+^ T cell populations to recombinant IL-35, both populations exhibited reduced cytotoxicity (143). Additionally, CD8^+^ T cells from patients with non-viral hepatitis-related hepatocellular carcinoma (HCC) showed a decrease in cytotoxic function when treated with recombinant IL-35 (144). Similarly, human breast cancer cells produce IL-35 which functions to convert conventional T cell populations to iTr35 cells (145). Together, this work supports a role for IL-35 in shifting the TME toward an immunosuppressive state (Figure 3C). Indeed, in a murine model of melanoma, IL-35 promotes T cell exhaustion within the TME by increasing expression of inhibitory receptors PD1, TIM3, and LAG3 (146). When IL-35 was neutralized within the tumor, T cell proliferation and activation were enhanced (146). Interestingly, IL-35 treatment of human pancreatic ductal adenocarcinoma (PDAC) cell lines induced ICAM1 expression indicating a role for IL-35 in tumor metastasis (147). In an orthotopic xenograft mouse model, overexpression of IL-35 contributed significantly to tumor establishment in an ICAM1-dependent manner (147).

The primary function of IL-35 within the TME appears to be in favor of a pro-tumor immune environment. In contrast to the work discussed above, Sun et al. outlined a potential anti-tumor role for IL-35 in NSCLC (148). In human patient samples, decreased IL-35 expression was observed in cancer sites compared to non-cancer sites. Through the use of NSCLC cell lines, this study demonstrated that IL-35 treatment resulted in suppressed beta-catenin expression and decreased migration, invasion, and proliferation of the cancer cells (148). Therefore IL-35 may behave similarly to IL-27 to have divergent roles in cancer progression that may be dependent on cancer type, stage, and TME landscape.

### Therapeutic Potential of IL-27, IL-30, and IL-35 Within the TME

The TME is home to many different immune cells that play critical roles in regulating tumor development, notably: myeloid derived suppressor cells (MDSCs), tumor associated macrophages (TAMs), cancer associated fibroblasts (CAFs), tumor associated neutrophils (TANs), T cells, and NK cells. To date, the role of this triumvirate of cytokines in the TME is not well understood, with IL-30 being the most understudied. Furthermore, IL-27 is the only cytokine of the three that is well-characterized and exhibits anti-tumoral properties. Below, we focus on the role of IL-27 and how its anti-tumor potential can be harnessed, as well as how neutralizing the apparent pro-tumor cytokines, IL-30 and IL-35, can further enhance an anti-tumor response in the TME.

MDSCs have immunosuppressive functions within the TME that make these cells a major obstacle for the success of cancer immunotherapies, therefore it is important to consider decreasing their activity in the TME. IL-27 can increase IRF8 expression, which negatively regulates MDSC development, by promoting apoptosis of myeloid cells and decreasing GM-CSF production, a critical cytokine for myeloid cell development (149). Furthermore, immunotherapies focused on inhibiting STAT3 have demonstrated success in preventing MDSC development, which may be attributed to the ability of STAT3 to downregulate IRF8 expression (150–152). Having greater STAT1 than STAT3 signaling can promote anti-tumor immune responses (68, 153). Therefore, it is possible that the addition of IL-27 alongside STAT3 inhibitors may serve to further enhance their efficacy, as inhibition of STAT3 could favor STAT1-dominant signaling, promoting the anti-tumor capacity of IL-27. The addition of IL-27 can promote CXCL10 production by MDSCs, resulting in the recruitment of anti-tumor NK and NKT cells reducing tumor size (154). In contrast, IL-35 can enhance MDSC accumulation resulting in suppression of anti-tumor immune responses (140). Therefore, it is possible that neutralizing IL-35 in the TME can reduce MDSC accumulation and favor an anti-tumor immune response.

Monocytic-MDSCs can differentiate into TAMs, which can be further classified as being M1 (classically activated; anti-tumor) or M2 (alternatively activated; pro-tumor) (100, 155). The majority of TAMs with an M2 phenotype are also PD-L1^+^ and exhibit an immunosuppressive capacity (156, 157). IL-27 has overlapping functions with IFN-γ (158) and may be able to promote M1 macrophage polarization while inhibiting M2 polarization (103). Furthermore, IL-27 is able to drive Th1 polarization and reduce Th2 polarization, which may also indirectly skew macrophage population to an M1 dominant subset within the TME. Whether IL-27 is able to activate tumoricidal macrophage functions while suppressing MDSC activity within the TME requires future investigation. TAMs can also secrete IL-35 resulting in tumorigenesis (141). This further highlights the importance of neutralizing IL-35 in the TME to favor tumor eradication.

CAFs secrete soluble factors in the TME generating cross-talk with immune cells and cancer cells, resulting in the simultaneous suppression of anti-tumor immune cells and activation of immune suppressive cells (159). Decreasing the proliferation of CAFs or decreasing their immunosuppressive secretory profile may prove to be an effective cancer therapy. In fact, IL-27 can suppress lung fibroblast proliferation by inhibiting TGF-β1 (160). The presence of IL-27 can also promote human lung fibroblast secretion of CXCL10 (161). It is possible that IL-27 may induce production of CXCL10 by CAFs, which may help with immune cell recruitment to the TME. Thus, IL-27 may serve to decrease fibroblast proliferation, while re-educating these cells to produce cytokines/chemokines and trigger an unfavorable environment for tumor cells.

TANs are generally recognized as being pro-tumor in the TME; however, because of their functional plasticity, TANs can take on pro- or anti-tumor states depending on the cytokine environment (162, 163). By inhibiting TGF-β, it may be possible to skew TANs to a more anti-tumor-like (N1) cell, compared to their pro-tumor counterpart (N2), which requires TGF-β (164). The ability of IL-27 to limit TGF-β may serve to promote N1 polarization, while IL-35 has demonstrated the capacity to promote N2 polarization favoring tumor progression (142, 160). Therefore, promoting IL-27 and reducing IL-35 functions on TANs needs further investigation for use as a potential immunotherapy.

To further promote tumorigenesis, the TME can prevent cytotoxic T cell and NK cell activation, while enhancing Treg activity. Using an adenovirus-vector secreting IL-27, Zhu et al. found that the presence of IL-27 in the TME enhanced T cell and NK cell recruitment, while subsequently depleting Tregs (99). This is further supported by research that outlines the role of gp130 signaling in reducing Helios, a transcription factor critical in Treg function (165). These studies outline how IL-27 can reduce Tregs, which may result in the loss of a cellular source of IL-35 leading to a potential anti-tumor immune response. Moreover, IL-27 can enhance cytotoxic anti-tumor responses of CD8^+^ T cells and NK cells, and therefore may be used to advance current T cell and NK cell therapies (86, 104–106, 166). The ability of IL-27 to increase CTL cytotoxicity while decreasing Treg activity, outlines the potential use of IL-27 in gene-therapy, cancer vaccines, T cell adoptive transfer, or CAR T cell therapies. In addition, use of IL-27 may enhance cytokine-mediated toxicity of NK cells. For example, the use of IL-15 can enhance NK cell cytotoxic function against ovarian cancer (167, 168) and IL-27 has been shown to enhance IL-15 mediated NK cell activation (169). This suggests that IL-27 should be further explored for use in combination with other cytokines to improve cytotoxic immune cell therapies.

As discussed above, IL-27 is endogenously produced as two separate subunits; however, IL-27p28 (IL-30) can signal alone resulting in tumor progression. Using a murine model, IL-30 conditional knockdown in prostate cancer stem cell-like cells resulted in decreased vascularization, and MDSC and Treg numbers, while increasing CTL presence in the tumor (170). Therefore, using therapies that inhibit the presence and/or activity of IL-30 within the TME may aid in tumor reduction. Alternatively, enhancing the probability of IL-27p28 binding with EBI3 to yield IL-27 would increase the abundance of IL-27 in the TME, while decreasing the availability of IL-30, further promoting IL-27 mediated anti-tumor immunity. Moreover, IL-27 can influence the availability of IL-35 and thus may affect the outcome of tumor survival and metastasis. Therefore, targeting different subsets of tumor associated cells by inhibiting IL-30 and IL-35, while promoting IL-27 signaling and production highlights the impact this cytokine triumvirate has on the fate of the TME.

## New IL-12 Family Members: IL-39 and IL-Y

IL-39, a complex of IL-23p19 and EBI3 subunits is thought to bind a receptor comprised of IL-23R and gp130 (171). IL-39 is expressed by activated B cells and promotes inflammation in a murine model of lupus (172). Recently, antibodies to IL-39 were shown to be effective in treatment of lupus (173). Interestingly, IL-39 has pro-tumorigenic effects in pancreatic cancer; in a human pancreatic cancer cell line, recombinant IL-39 treatment resulted in decreased p21 mRNA expression, enhancement of proliferation, and decreased apoptosis (174). However, little is known about the biology of this cytokine and its role in other cancers.

IL-Y is a synthetic complex of p28 and p40 subunits, and is thought to bind to a receptor comprised of WSX-1 and IL-12β1 (171). In experimental settings, IL-Y is generally synthesized with the two subunits connected with a linker sequence, and it is commercially available as such. So far, IL-Y has been examined in therapeutic models. In a model of experimental autoimmune uvetis (EAU), injection of IL-Y, created as a fusion protein, inhibited Th1 and Th17 differentiation and function, thereby suppressing EAU (175). Interestingly, p40 alone inhibited Th1 differentiation and function, but p28 alone inhibited Th17 differentiation and function. Injection of the p40 subunit did impact EAU, however injection of p28 alone also suppressed EAU. Together, this indicates the individual functions of p28 and p40 with respect to Th differentiation are not compromised upon the formation of IL-Y. Moreover, the p28 component of IL-Y may be responsible for IL-Y-mediated EAU inhibition. In a diabetic murine model, use of an adenovirus vector expressing IL-Y decreased expression of regulators of inflammation, such as IFN-γ, which resulted in prevention of onset of hyperglycemia (176). Moreover, IL-Y also prevented anti-tumor responses in a murine model of fibrosarcoma and resulted in increased tumor growth *in vivo* (176). Given the novel uses of IL-Y, it is possible that synthetic combinations of the IL-27-IL-30-IL-35 cytokine subunits may be utilized to direct immune cell activities in the TME in novel immunotherapeutic cancer treatments targeted toward enhancing anti-tumor immune responses, while directly targeting the tumors themselves.

## Interplay Between IL-27, IL-30, and IL-35 Subunits

As discussed above, commercially available recombinant IL-27, IL-35, IL-39, and IL-Y may be produced with a peptide linker chain between the two subunits to prevent dissociation, allowing for the study of the specific heterodimeric cytokines (Figure 3A). However, it should be noted that results from subunit deficient mice should be considered carefully (Figure 3B). For example, deficiency in EBI3 will impact IL-27 and IL-35 expression as well as the newly defined IL-39. Similarly, use of p28 deficient mice not only knocks out IL-27, but also IL-30. Several studies using p28 knockout animals perform control experiments whereby recombinant IL-27, containing a linker between the EBI3 and p28 subunits to prevent dissociation, is injected into p28 deficient mice to establish specificity of IL-27 vs. IL-30. Similar experiments should be considered in the use of EBI3 deficient animals as well. Furthermore, it is not known if, upon secretion, the IL-27 or IL-35 subunits could dissociate, leading to the formation of IL-30, IL-39, or other as of yet unidentified cytokines. The somewhat promiscuous use of receptor chains by IL-27-IL-30-IL-35 also adds complexity in teasing out the functional requirements of each cytokine. Studies in models deficient in WSX-1 or gp130 would indicate that one or more of the three cytokines is unable to function, thus obfuscating the interpretations of such models. Furthering our understanding in this regard is critical to defining the functions of these cytokines in the TME.

## Conclusion

Interactions between IL-27, IL-30, and IL-35 subunits within the TME may influence tumor development and may contribute to immunotherapeutic outcomes. More research is needed to uncover the individual and unique functions of each cytokine within the TME and their potential positive or inhibitory effects on both immune and cancer cells. When using models to investigate the interactions at play within this group of cytokines it is important to consider that neutralization or knockout of cytokine subunits or their receptors may affect more than one cytokine. For example, knockout of p28 has the potential to affect not only IL-30 activity but also p28/CLF, p28/sIL-6Rα, and IL-27 activities. Furthermore, the promiscuous use of receptor chains by this cytokine triumvirate combined with the potential recombination between cytokine subunits adds to the complexity of interpreting data from knockout or neutralization models. The presence of the artificially added linker sequences in recombinant and transduced IL-27 and IL-35 is important to consider when studying the potential interplay between these cytokines as it impedes the potential formation of IL-30 via dissociation, which would also impact formation of p28 complexes. Understanding the mechanisms of these three cytokines and their complex roles within the TME and the immune landscape is critical for developing therapeutics that promote anti-tumor responses by influencing dynamics between IL-27, IL-30, and IL-35.

## Author Contributions

OK, KS, NO, SB, and KG contributed to the conceptualization, discussion, and writing of this review. OK, KS, and KG designed and composed the figures.

### Conflict of Interest

The authors declare that the research was conducted in the absence of any commercial or financial relationships that could be construed as a potential conflict of interest.
